# Design, Fabrication, and Evaluation of Multifocal Point Transducer for High-Frequency Ultrasound Applications

**DOI:** 10.3390/s19030609

**Published:** 2019-02-01

**Authors:** Thanh Phuoc Nguyen, Nguyen Thanh Phong Truong, Nhat Quang Bui, Van Tu Nguyen, Giang Hoang, Jaeyeop Choi, Thi Tuong Vy Phan, Van Hiep Pham, Byung-Gak Kim, Junghwan Oh

**Affiliations:** 1 Interdisciplinary Program of Biomedical Mechanical and Electrical Engineering, Pukyong National University, Busan 48513, Korea; ntphuoc2000@gmail.com (T.P.N.); phongtruongbk@gmail.com (N.T.P.T.); nguyen.vantu91@gmail.com (V.T.N.); eve1502@naver.com (J.C.); phanvy120690@gmail.com (T.T.V.P.); pvhiep.mta.hut@gmail.com (V.H.P.); 2 Center for Marine-Integrated Biomedical Technology, Pukyong National University, Busan 48513, Korea; nhatquang85@gmail.com (N.Q.B.); giang.hoanggiang244@gmail.com (G.H.); 3 College of Future Convergence, Pukyong National University, Busan 48513, Korea; 4 Department of Biomedical Engineering, Pukyong National University, Busan 48513, Korea

**Keywords:** ultrasonic transducer, multifocal point transducer, high-frequency applications, high-frequency ultrasound transducer, PVDF focused transducer

## Abstract

The present study illustrates the design, fabrication, and evaluation of a novel multifocal point (MFP) transducer based on polyvinylidene fluoride (PVDF) film for high-frequency ultrasound application. The fabricated MFP surface was press-focused using a computer numerical control (CNC) machining tool-customized multi-spherical pattern object. The multi-spherical pattern has five spherical surfaces with equal area and connected continuously to have the same energy level at focal points. Center points of these spheres are distributed in a linear pattern with 1 mm distance between each two points. The radius of these spheres increases steadily from 10 mm to 13.86 mm. The designed MFP transducer had a center frequency of 50 MHz and a −6 dB bandwidth of 68%. The wire phantom test was conducted to study and demonstrate the advantages of this novel design. The obtained results for MFP transducer revealed a significant increase (4.3 mm) of total focal zone in the near-field and far-field area compared with 0.48 mm obtained using the conventional single focal point transducer. Hence, the proposed method is promising to fabricate MFP transducers for deeper imaging depth applications.

## 1. Introduction

Recently, high-frequency ultrasound transducers have been widely used in various biomedical applications including intravascular, skin, and eye imaging on animals [1,2,3]. A recent study developed an intravascular photoacoustic imaging method using the ultrasonic transducer and multimode fiber to identify atherosclerotic plaques through spatial and functional information of transportation of light in tissues [4]. It is a well-known phenomenon that for developing a high-quality deep scanning transducer, the length of the depth of field (DOF) or focal zone should be deeper. Therefore, several research teams have concentrated on developing focused transducers with the aim to create more effective devices [1,2,3,5,6,7,8,9,10,11,12,13,14,15].

The parameters of the focused transducers including frequency, focal length, aperture, spatial resolution and the DOF or focal zone should be appropriately designed to achieve the best image quality. DOF or focal zone is defined as the area around the focal point within the transmitted sound beam where the beam diameter narrow to its minimum size. To obtain an ultrasound image, several well-established scanning methodologies such as B-scan and C-scan modes have already been discussed by several researchers [1,5,6,8,16]. The following B-scan and C-scan modes are associated with the movement of the transducer across the target using two motors in the X-Y plane. To achieve the best image quality, the target must be placed in the DOF of the transducer. However, this is challenging in B-scan mode for larger objects with a smaller DOF of the transducer. Additionally, short DOF causes the reducing the signal-to-noise ratio (SNR) in the far-field [17]. Several researchers have proposed numerous methods to increase the DOF of transducers [5,9,17,18,19]. Jeffrey et al. [5] have proposed a complex imaging procedure based on a PVDF annular array transducer. Although the annular array transducer has been reported to have an ideal geometry for increasing the DOF up to 6 mm, it requires a complicated fabrication process and a more sophisticated system because of the small width of elements. Moreover, to achieve the final image from the scanning of the annular array transducer, data is acquired from individual transmit/receive annuli pairs. Thereafter, the image was reconstructed using the algorithms of digital synthetic aperture.

Another scanning method, D-scan or “Depth-scan” has been developed in recent decades to obtain images at different depths of tissues or targets [18]. Based on the mechanical movement, transducers are moved in the depth direction and the short B-scan is performed several times. The challenging task in this scanning procedure is “B/D-scan” which effectively compose all scans to obtain a high-quality image. Using a fixed focused transducer, its image quality is significantly deteriorated in cases wherein the target is located outside the focus area. To solve this problem, the adaptive synthetic aperture focusing technique (SAFT) was introduced to extend the DOF and significantly improve the resolution of the image quality [20]. However, this method achieved a low SNR.

To overcome the above issues, the researchers developed a novel MFP transducer for extending DOF without applying array transducers or performing D-scan. In contrast to conventional transducers, the proposed MFP transducer could generate multifocal points (MFPs) in the axial direction and receive the excitation pulse simultaneously. The MFP transducer could provide multi-focused depths to improve DOF. The authors have developed a 50-MHz MFP transducer with five focal points. The outer aperture diameter of the fabricated transducer is 8.84 mm, with 1 mm distance between two focal points. The fabrication method is briefly described in Section 2.3. 

In the present study, the design, fabrication, and evaluation of a PVDF-based MFP broadband-focusing transducer is reported. Two types of transducers, such as a single focal point (SFP) and MFP, were fabricated and tested using the pulse-echo response method. A wire phantom experiment was conducted to demonstrate the effectiveness of the MFP transducer as compared with the SFP transducer. It is worth noticing that the DOF of five-focal point transducer in this present study is 4.3 mm, for which only a simple method for image scanning is required. In the previous study [19], the DOF of eight-element kerfless annular array transducer was 4.5 mm. To obtain the image of four-wire of phantom; however, the system performed eight scans (eight channels) to record all data. The method presented in this study is simpler to extend the DOF for deeper image applications.

## 2. Design and Fabrication of Multifocal Point Transducers 

### 2.1. Materials

As the key component of ultrasonic transducer, certain piezoelectric materials including lead zirconate titanate (PZT) [21,22,23,24], lead niobiumzine zirconate titanate (PMN-PT) crystal [1,21,22,25,26,27], lithium niobate (LiNbO_3_) single crystal [2,3,13,28,29], zinc oxide (ZnO) [12,28], and polyvinylidene fluoride (PVDF) film [3,5,6,9,30] have been extensively investigated and reported. The interesting properties of these piezoelectric materials including electromechanical coupling coefficient, acoustic impedance, dielectric constants, piezoelectric coefficients, and sound velocity have also been reported. 

In the present work, PVDF (polyvinylidene fluoride) was selected for the fabrication of MFP transducer because of its advanced multi-material properties. Although the acoustic impedance (~4 MRayl) of PVDF film is lower than piezoceramics and crystal materials, PVDF exhibits excellent mechanical flexibility that helps this piezopolymer to be easily tailored and pressed into a curved shape. Typical properties of PVDF are listed in Table 1. Moreover, because PVDF film has a relatively high insertion loss, their acoustic impedance is more easily matched to human tissues (~1.5 MRayl) compared with that of PZT and LiNbO_3_ materials. Additionally, PVDF film reveals a low dielectric constant that is suitable for electrical impedance matching [29]. PVDF has the widest bandwidth among the above-mentioned materials that can be used to generate high-resolution ultrasonic images. 

### 2.2. Transducer Design

To extend the focal zone of the focused transducer, it is essential to determine the parameters that have effects over the focal zone. These parameters for PVDF transducer were designed using a Krimholtz–Leedom–Matthaei (KLM) model-based simulator. The profile of the developed MFP transducer could be determined from the KLM simulation. 

The parameters of the focused transducer are listed below:

(1) $$f_{\#}=\frac{R}{D}$$

(2) $$N=\frac{D^{2}f_{c}}{4c}$$

(3) $$\;S_{F}=\frac{R}{N}$$

(4) $$F_{Z}=S_{F}^{2}\frac{2N}{1+0.5S_{F}}$$

(5) $$\delta_{L}=1.02c\frac{f_{\#}}{f_{c}}$$

(6) $$\delta_{A}=\frac{SPL}{2}$$

where, *R* is the focal length, *D* denotes the aperture diameter, *f_#_* stands for f-number, *c* is the speed of sound in load medium, *f_c_* is the center frequency, $\delta_{L}$ denotes the lateral resolution, $\delta_{A}$ is axial resolution, *SPL* explains the spatial pulse length, *N* represents the near-field area, *S_F_* is the normalized focal length, and *F_Z_* denotes the focal zone.

#### 2.2.1. Pulse-Echo and Frequency Spectra Simulation

**Figure 1:**
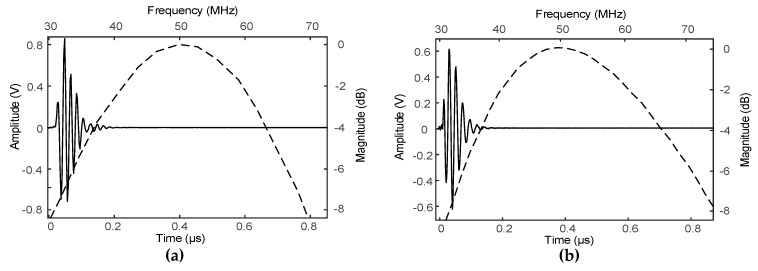
The simulated pulse-echo response (solid line) and their frequency spectra (dashed line) for 50 MHz PVDF of (**a**) single-focal point transducer and (**b**) the second part of multifocal point transducer. The simulated pulse-echo response and frequency spectra of the first part were similar to the signals of single-focal point transducer, whereas the other parts were similar to the second part.

Figure 1 shows the BioSono KLM simulation results. The center frequency and −6 dB bandwidth of the SFP transducer were designed as proposed and were 50 MHz and 66%, respectively, and those of the MFP transducer were 50 MHz and 68%. 

From these Equations (1)–(4), for the SFP transducer at the center frequency of 50 MHz, the focal length of 12.7 mm, the aperture diameter of 9 mm, and propagation speed of water is 1540 m/s, the focal zone was calculated as 0.48 mm, and the lateral resolution was 44 μm. To effectively extend this focal zone, the authors designed the MFP transducers that have multiple focal zones. These focal zones were slightly overlapped with each other to generate continuous focus depths. 

#### 2.2.2. Design of the Multifocal Point Transducer

**Figure 2:**
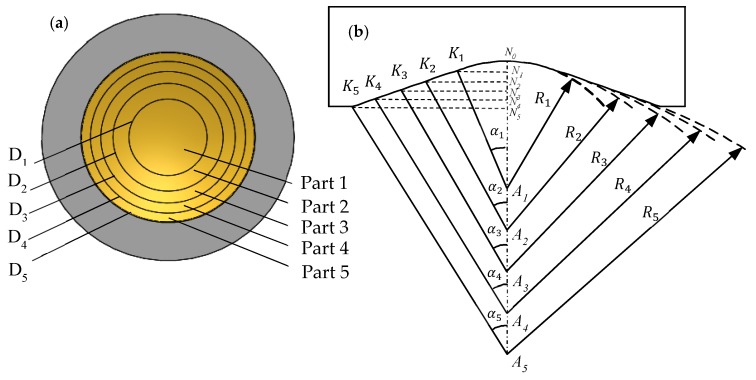
(**a**) The front face of MFP transducer is constructed from five equal area parts of five different spheres. (**b**) The profile of MFP transducer surface.

To obtain focused images at varying depths, the main challenges are to develop the MFP transducer using five focal points. These focal points were distributed in the axial direction with an interval of 1 mm (b = 1 mm). The front face of the MFP transducer was divided into five parts with an equal area. Each part comprised a spherical surface with a radius “Ri” and a height “hi”, respectively. Figure 2 shows the structure of the multi-spherical profile. 

The surface parameters of the MFP transducer are defined as follows:

- The radius of the spheres (focal length): R_1_ = A_1_N_0_ = A_1_K_1_, R_2_ = A_2_K_2_ = A_2_K_1_, R_3_ = A_3_K_3_ = A_3_K_2_, R_4_ = A_4_K_4_ = A_4_K_3_, R_5_ = A_5_K_5_ = A_5_K_4_
- The aperture diameter of each part: $D_{i}=2N_{i}K_{i}=2R_{i}\sin\alpha_{i}$, where, i = 1 to 5.
- The height of the spherical part: $h_{i}=N_{i}N_{i-1}$, where, i = 1 to 5.
- The distance between two closed focal points: $A_{i}A_{i+1}=b$, where, i = 1 to 4.

From Figure 2, the specifications are designed as: b = 1 mm, R_1_ = 10 mm, R_2_ = 10.98 mm, R_3_ = 11.95 mm, R_4_ = 12.91 mm, R_5_ = 13.86 mm, D_1_ = 4 mm, D_2_ = 5.62 mm, D_3_ = 6.88 mm, D_4_ = 7.92 mm, D_5_ = 8.84 mm, h_1_ = 0.2 mm, h_2_ = 0.18 mm, h_3_ = 0.16 mm, h_4_ = 0.15 mm, h_5_ = 0.14 mm, $\alpha_{1}=11.53^{{}^{\circ}},\;\alpha_{2}=14.86^{{}^{\circ}},\alpha_{3}=16.73^{{}^{\circ}},\alpha_{4}=17.87^{{}^{\circ}},\alpha_{5}=18.59^{{}^{\circ}}.$

**Figure 3:**
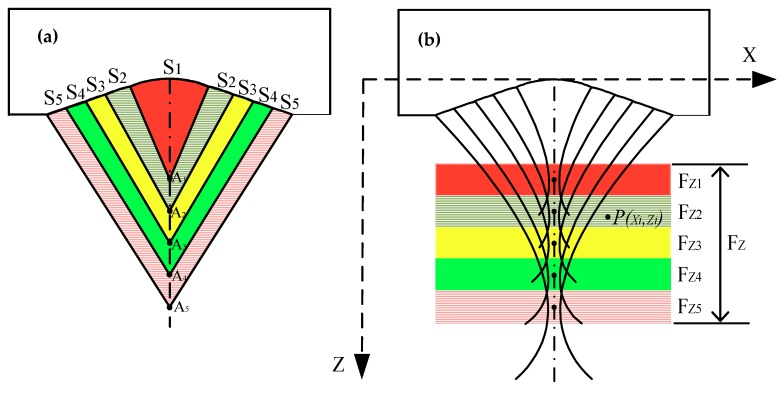
(**a**) Distribution of multifocal point, (**b**) distribution of focal zones. The point P(x_i_, z_i_) is located in the focal zone will get the good image. The x_i_ represents the distance in the X-axis, z_i_ is the depth of point P in the Z-axis.

The length of the focal zone of MFP transducer can be extended by connecting several focal zones. These focal zones were generated using several different spheres and their center points (A_i_, I = 1 to 5) are located on a straight line. Each spherical shape contributes to one focal point as shown in Figure 3 a. The transducer can be moved along with the X-axis to acquire the data. When a point P (xi, zi) is positioned in the acoustic field of the sphere “S_i_” (“S_i_” is called part “i”, i = 1 to 5), the detected signal should contribute to the focal point A_i_. The focal length of part “i” is “R_i_ “. Each part of the multifocal sphere can detect the signal in each focal zone, which is described in Figure 3b. For example, the part “i” can detect the signal in the focal zone “F_zi_”. The object located around the point A_i_ in the focal zone “F_zi_” will acquire the best image. The target located outside of the focal zone will obtain the blurry image. 

The sphere 1 (part 1) was unable to capture a good image at point P with z_i_ > R_1_. As a result, other spheres were designed with different focal depths to acquire a good image for long depth objects. The total length of the focal zone (F_z_) for this transducer is the sum of all individual focal zone components (F_zi_). To avoid the gap between the adjacent focal zones, each focal zone and distance between two focal points were carefully calculated. 

The parameters of each part in Figure 3b were calculated using Equations (1)–(4) (Table 2). Therefore, the total DOFs for MFP transducer is the sum of the focal zones (4.3 mm). 

**Figure 4:**
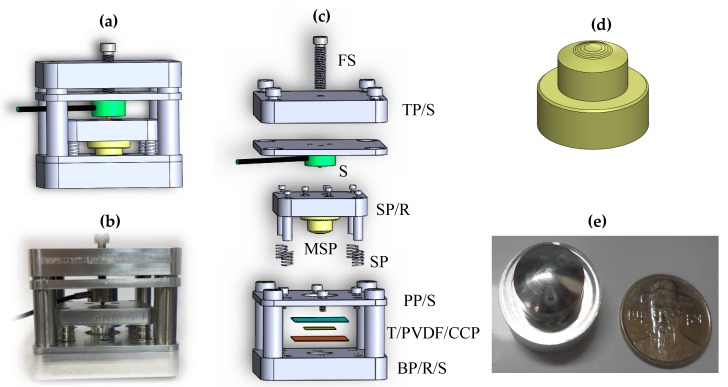
(**a**) Design of the press-fit system for creating the front face of the multi-focal point (MFP) transducer. (**b**) Photograph of the press-fit system. (**c**) Components of the press-fit system: Base plate/rod/screw (BP/R/S), Teflon/PVDF film/copper-clad polyimide (T/PVDF/CCP), pressure plate/screw (PP/S), spring (SP), multi-spherical pattern (MSP), slide plate/rod (SP/R), sensor of force (S), top plate/screw (TP/S), force screw (FS). (**d**) Design of multi-spherical pattern for creating transducer using five focal points. (**e**) Photograph of multi-spherical pattern.

Using Solidworks software (Version. 2016, Dassault Systemes, USA) a press-fit system (Figure 4a) and a multi-spherical pattern (Figure 4d) were designed to form a multi-spherical shape for the active element. CNC machine was used to fabricate the press-fit components (Figure 4b) and the multi-spherical pattern (Figure 4e).

### 2.3. Transducer Fabrication

To demonstrate the strong features of the MFP transducer, two types of 9 μm PVDF focused transducers (i.e., MFP and SFP transducers) were developed and fabricated using the press-focused method. The fabrication method for the SFP transducer in the present work is similar to that described previously [6]. A steel ball bearing was used to form 12.7 mm of radius for a single-spherical shape of the active element. 

The fabrication process for the MFP transducer was divided into two stages. In the first stage, a multi-spherical shape for the active element was formed using the press-fit system (Figure 4b). The copper-clad polyimide (CCP; Hanwha Corp., FCCL, Seoul, Korea), PVDF film (Piezotech S.A.S, Pierre Benite, France) and Teflon films (the size of 4 ×4 cm) were prepared as described previously [6]. The PVDF and CCP were bonded together using a single drop of epoxy (EPO TEK 301, Epoxy Technology, Billerica, MA, USA). The Teflon film was placed on the PVDF surface to protect the surface while pressing the spherical pattern in the press-fit system to avoid tearing the membranes. Three films were placed on the base plate surface at the center hole. The base plate was attached with four rods to form the standard assembly. The pressure plate was placed on these films through the four rods and clamped with the base plate using four screws. The slide-plate, which had been attached to the multi-spherical pattern (Figure 4e), was connected to these four rods to ensure the concentricity of the holes from these plates. The springs were used to reduce the vibration during the pressing focus. The tension of the active element surface was managed to optimize the force value from the force sensor. The top plate was fixed to the below plate using screws. The display unit is connected to both force sensor and the power supply. To form the concave shapes of the transducer, a hexagon bar wrench was used to rotate the forcing screw which makes a uniform pressure on force sensor and multi-spherical pattern. 

Following the pressing of these films, the press-fit system was inverted and the Teflon tube was inserted into the center hole of the base plate. To maintain the spherical shape of the PVDF membrane after curing, the nonconductive epoxy was filled into the Teflon tube. The press-fit system was heated in an oven at 65 °C for 2 h. Thereafter, the press-fit system was disassembled to take the acoustic stack that included an epoxy plug with the CCP and PVDF films attached to it. Finally, the Teflon film was removed out from the acoustic stack. 

**Figure 5:**
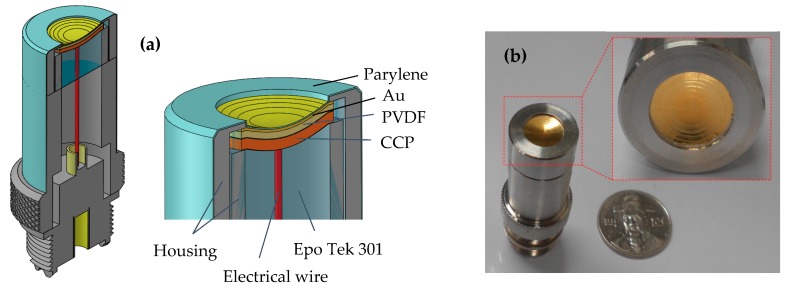
(**a**) A cross-sectional view of MFP transducer. (**b**) Photograph of the fabricated MFP transducer for long depth ultrasound images.

The second stage involved the fabrication of the acoustic stack with transducer housing. The PVDF and CCP were trimmed as close as possible to the epoxy plug. A small line of CCP was kept and soldered using an electrical wire, which was then connected to the center pin of the UHF connector. The acoustic stack was placed concentrically with transducer housing. A piece of PVDF was connected to housing using silver epoxy (H20 epoxy, Epoxy Technology, Inc., USA) to form a ground path. To maintain the long-term electrical and mechanical stability of the transducer, a nonconductive epoxy was filled into the open space inside housing. Following the curing of the epoxy, a negative electrode of the active elements was connected to the UHF connector through the housing transducer. An electrical impedance matching was placed inside the housing, followed by a nonconductive epoxy filling. A quarter-wavelength thickness of parylene was sputtered on the front face of the transducer to protect the surface and function of the matching layer, which ensured the transfer of acoustic energy between the piezoelectric and the load medium. Figure 5a shows the cross-sectional view of the MFP transducer with the components used for fabrication. The digital photograph of the completed MFP transducer is shown in Figure 5b. 

## 3. Performance Evaluation and Discussion 

### 3.1. Experimental Setup 

**Figure 6:**
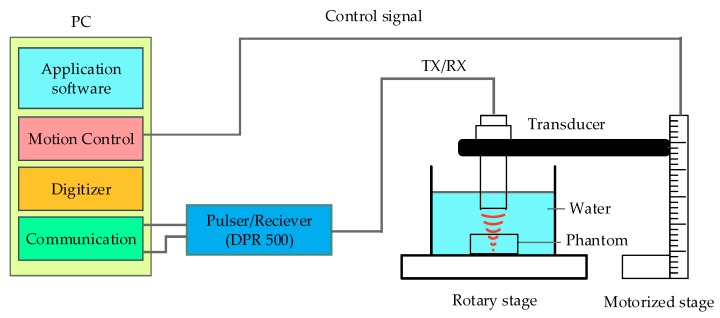
The schematic diagram of the experiment for the wire phantom using MFP transducer.

Figure 6 shows the schematic representation of the experimental procedure. The transducer was connected to a computer-controlled remote (DPR 500, JSR Ultrasonics, Pittsford, NY, USA) pulser/receiver and excited using an electrical impulse of 200 Hz repetition rate at 50 Ω damping and 3 μJ energy per pulse. To measure the pulse-echo and frequency spectra of transducers, a glass plate was placed at the focal point as a target. The reflected waveform was received using a 500 MHz bandwidth receiver with a high pass filter of 5 MHz and a low pass filter of 500 MHz. The obtained raw data was further digitized at a high-speed sampling frequency of 500 Megasample/s. The echoes were digitized by an 8-bit digitizer (NI PCI-5153EX, National Instruments, Austin, TX, USA). 

The movement of the transducer was controlled using stepper motor (UE63PP, Newport Corporation., CA, USA) and driven using Universal motion controller/driver (ESP300, Newport Corporation., CA, USA). A LabView (LabView 2014, National Instrument, Austin, TX, USA) program was developed to control all the process mentioned above. A computer-controlled scanning stage was moved along with the X-axis to obtain B-scan image and in the X-Y plane to obtain C-scan image. 

**Figure 7:**
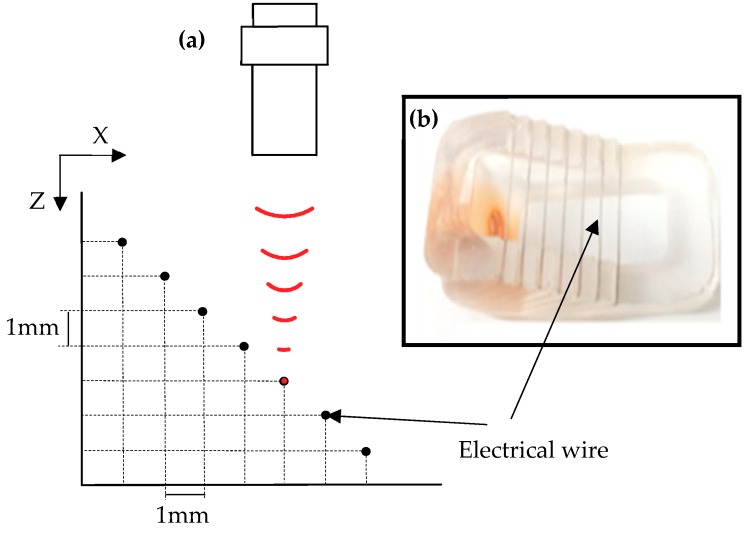
(**a**) The schematic of wire phantom test. (**b**) The photograph of wire phantom.

Seven phantom wires (25 µm) were positioned diagonally with an equal distance of 1 mm in the axial direction and 1 mm in the lateral direction (Figure 7a). The wire was placed at the focal point of the transducer in degassed water and scanned in a lateral direction. The echo signal that reflected from the wire was used to build the beam profile to establish the size of the beam in the lateral direction. Data were imported to MATLAB software (Version. 2013a, Mathworks, Natick, MA, USA) for image processing. 

### 3.2. Results and Discussions

**Figure 8:**
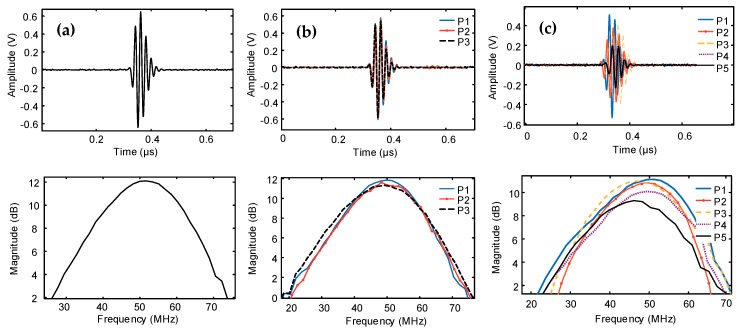
Measured time domain (top row), pulse-echo response (bottom row) of (**a**) single-focal point transducer T_1_, (**b**) three-focal points MFP transducer T_2_, and (**c**) five-focal points MFP transducer T_3._

Pulse-echo experiments were employed in a water tank using a glass plate placed at the acoustic focus. The pulse-echo response and the frequency spectrum of transducers are shown in Figure 8.

Table 3 summarizes the comparison of simulated and measured characteristics of three fabricated transducers. Transducer T_1_ is the SFP transducer, T_2-Pi_ is part “i” (i = 1 to 3) of the MFP transducer T_2_, and T_3-Pj_ are reported as part “j” (j = 1 to 5) of the MFP transducer T_3_. In general, the experimental measurements were consistent with the simulation results. The KLM model predicted the center frequency a slightly different to the observed values. For the bandwidth, the majority of experimental values are somewhat higher than expected from the simulation. Specifically, the KLM model expected 68% bandwidth for the MPF transducer T_3_ while detected bandwidths were 66–70%, which are not much different from simulation. The T_3-P5_, which is the part 5 (ring 5) of the transducer T_3_, has the largest aperture size (8.84 mm) and the widest bandwidth (70%) as compared to the others.

In terms of the measured center frequency (f_c_), the SFP transducer T_1_ yielded the highest value, and those of two MFP transducers T_2_ and T_3_ were slightly lower than the simulation. For the bandwidth measured at −6 dB, transducer T_3_ exhibited a narrower bandwidth compared to the simulation, whereas the bandwidth of the two transducers T_1_ and T_2_ were wider than the simulations. Although the same type of 9 µm PVDF film is used to generate all transducers in this study, the measurement of center frequencies and the bandwidths were different from the models due to their aperture sizes changing. 

The images of the wire phantom were acquired using both SFP and MFP transducers. Their DOFs were assessed and compared. B-scan and C-scan images of the SFP transducer were employed as a reference to evaluate the performance of the MFP transducer. 

**Figure 9:**
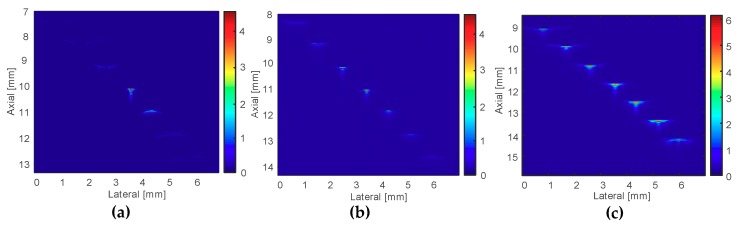
B-scan images of wire phantom for (**a**) single-focal point transducer T_1_, (**b**) three-focal points MFP transducer T_2_, and (**c**) five-focal points MFP transducer T_3_.

Figure 9 shows the images of the B-mode scans using three transducers T_1_, T_2_, and T_3_. The wires, which were placed in the focal zone of the transducers, have acquired the bright points in the image; otherwise, they displayed blurred points in images. We observed that only the MFP transducer T_3_ exhibited seven bright points, indicating a long focal zone for deeper images. 

**Figure 10:**
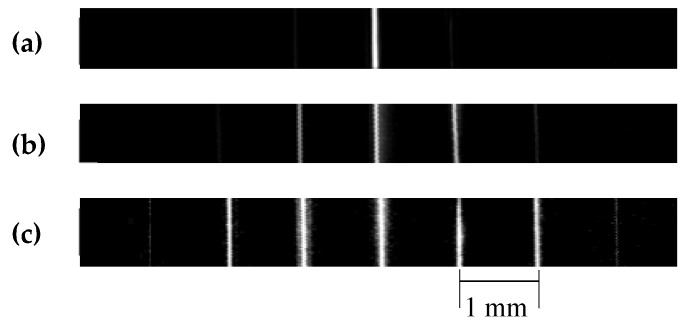
C-scan images of the wire phantom of (**a**) single-focal point transducer T_1_, (**b**) multifocal point transducer with three focal points T_2_, and (**c**) multifocal point transducer with five focal points T_3_.

Figure 10a shows the C-scan image using the SFP transducer T_1_. Because the focal zone of T_1_ was 0.48 mm, and the distance between two wires was 1 mm, the acquired image displayed only the wire at the focal point. Figure 10b revealed the image obtained from the MFP transducer T_2_ using three focal points. The image displayed only three wires on the middle of the wire phantom model. Because of the smaller focal zone of about 3 mm (T_2_), the other wires were out of focus and could not be captured clearly. On the contrary, the MFP transducer T_3_ using five focal points created a clear image of five wires at the center (Figure 10c). It is worth noticing that distance from the second wire to the sixth wire was 4 mm and the focal zone of T_3_ was 4.3 mm. Therefore, the five center wires placed in the focal zone were imaged brighter than the two wires outside the focal zone.

**Figure 11:**
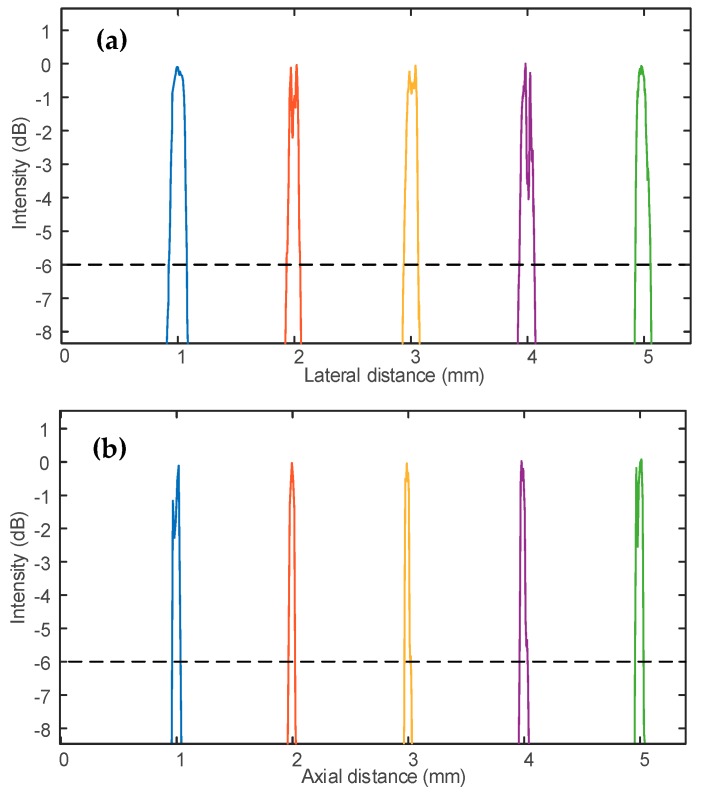
The measured resolution of the MFP transducer T3 with five focal points. (**a**) Lateral brightness profile. (**b**) Axial brightness profile.

As shown in Figure 11, the resolution of the MFP transducer T_3_ using five focal points were measured from the B-scan mode at focus depths of the transducer.

The wire target was used to calculate the pulse intensity integral. Lateral resolutions of five focal points were determined by the full width at half maximum (FWHM, −6 dB) of the corresponding cross-sectional profile and wire diameter. These lateral resolutions were measured to be 156 µm, 108 µm, 113 µm, 135 µm, and 130 µm respectively for these points. The axial resolutions at five focal points detected by the full width at half maximum (FWHM, −6 dB) were 66 µm, 61 µm, 61 µm, 65 µm, and 66 µm respectively. The fabrication skill of the MFP transducer also affected the transducer quality, such as frequency, noise, resolution, and amplitude of the pulse-echo response. 

## 4. Conclusions

This study reported a novel design, fabrication, and characterization of MFP transducers that significantly increased the focal zone (4.3 mm) compared with that generated by a SFP transducer (0.48 mm). The clear image of five phantom wires has demonstrated the extended focal zone of the proposed MFP transducer. It also shows the capability of extending the focal zone for a larger size of the target being imaged without the necessity of applying depth scans or any complex SAFTs. It is noted that the eight-element kerfless annular array transducer in [19], which has a DOF of 4.5 mm with eight-element (eight-ring), requires a complex scanning method to obtain the final image. Meanwhile, the five-focal (five-ring) transducer in this present study, which has a DOF of 4.3 mm, requires only a simple method to scan the image. Compared with the SFP transducer, the developed MFP transducer yielded a consistent image quality for a much larger excitation depth. The method developed in the present report can be promisingly used to expand a larger focal zone by increasing the number of focal points of a transducer. Specifically, the proposed five-focal point transducer, is capable of simultaneously generating five focal zones in the axial direction. Therefore, MFP transducers have a great potential for imaging in long DOF applications.

## Figures and Tables

**Table 1:** Typical properties of PVDF ^1^.

Property	Value
Electromechanical coupling coefficient (K_t_)	0.12–0.15
Molecular formula	(CH_2_CF_2_)
Relative clamped dielectric constant ($\varepsilon^{S}/\varepsilon_{0}$)	11
Mechanical quality factor (Q_m_)	~20
Density (kg/m^3^)	1800
Longitudinal wave velocity (m/s)	2110
Acoustic impedance (MRayl)	3.9
Curie temperature (°C)	100
Melting temperature (°C)	160–180

^1^ Data reported by Piezo film sensor, AMP Inc, Valley Forge, PA.

**Table 2:** The estimated parameters of the MFP transducer using five focal points (distance between two focal points is b = 1 mm; center frequency of each part is f_c_ = 50 MHz).

Parameter	1st Part	2nd Part	3rd Part	4th Part	5th Part
Focal depth (mm)	10	10.98	11.95	12.91	13.86
Aperture diameter (mm)	4	5.62	6.88	7.92	8.84
f_#i_	2.5	1.94	1.73	1.62	1.56
F_zi_ (mm)	1.48	0.91	0.73	0.64	0.59

**Table 3:** Comparison between simulated and measured results.

Transducer	Simulated (KLM)		Measured results	
	f_c_ (MHz)	BW (%)	f_c_ (MHz)	BW (%)
T_1_ (SFP)	50	66	51	68
T_2-P1_ (MFP)	50	68	50	68
T_2-P2_ (MFP)			49	69
T_2-P3_ (MFP)			49	72
T_3-P1_ (MFP)	50	68	50	66
T_3-P2_ (MFP)			49	67
T_3-P3_ (MFP)			47	68
T_3-P4_ (MFP)			50	68
T_3-P5_ (MFP)			48	70
